# Oculomotor Nerve Palsy due to an Odontogenic Abscess Originating from the Mandibular Third Molar

**DOI:** 10.1155/2012/147628

**Published:** 2012-12-31

**Authors:** Hee-Keun Park, Moon-Key Kim, Sang-Hoon Kang

**Affiliations:** ^1^Department of Oral and Maxillofacial Surgery, National Health Insurance Corporation Ilsan Hospital, 100 Ilsan-ro, Ilsan-donggu, Goyang, Gyeonggi-do 410-719, Republic of Korea; ^2^Department of Oral and Maxillofacial Surgery, College of Dentistry, Yonsei University, 134 Shinchon-Dong, Seodaemun-Gu, Seoul 120-752, Republic of Korea

## Abstract

We report a case of oculomotor nerve palsy (ONP) as a result of odontogenic infection originating from the third molar, which is considered rare.

## 1. Introduction

Pathological lesions, trauma, and infection can cause palsy of the oculomotor nerve, the third cranial nerve [1]. We report a rare case of oculomotor nerve palsy (ONP) as a result of odontogenic infection originating from the third molar.

## 2. Case Presentation

**Figure 1:**
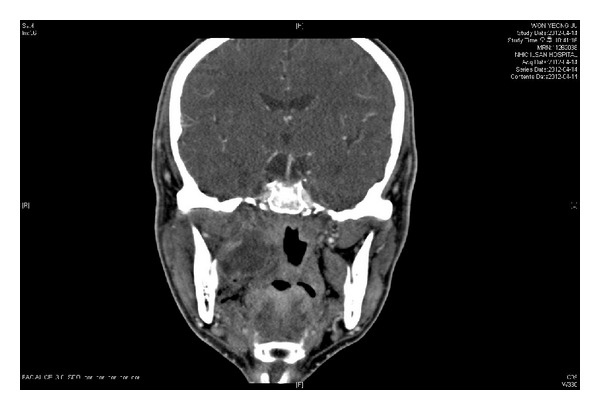
On CT images, an abscess pocket had diffused from the right pterygomandibular area to the right cavernous sinus area.

A 57-year-old male visited the hospital for swelling of the right mandibular area and trismus that had persisted for 7 days. The patient's medical history indicated diabetes mellitus as well as habitual smoking and alcohol drinking. Physical examination revealed redness, swelling, and fluctuation around the right mandibular third molar and pterygomandibular area. An abscess pocket was detected by computed tomography (CT) in the right pterygomandibular space (Figure 1). Immediate intravenous antibiotic treatment was started. Drainage of pus by an intraoral incision under local anaesthesia was planned; however, the patient complained of sudden blepharoptosis, difficulties in eye raising, and blurred vision in his right eye. Abduction was the only possible movement of the right eye in ocular findings. No double vision or pain in the orbital area was reported, and neurological evaluation revealed pupil dilation and blepharoptosis, indicating that right ONP had occurred.

**Figure 2:**
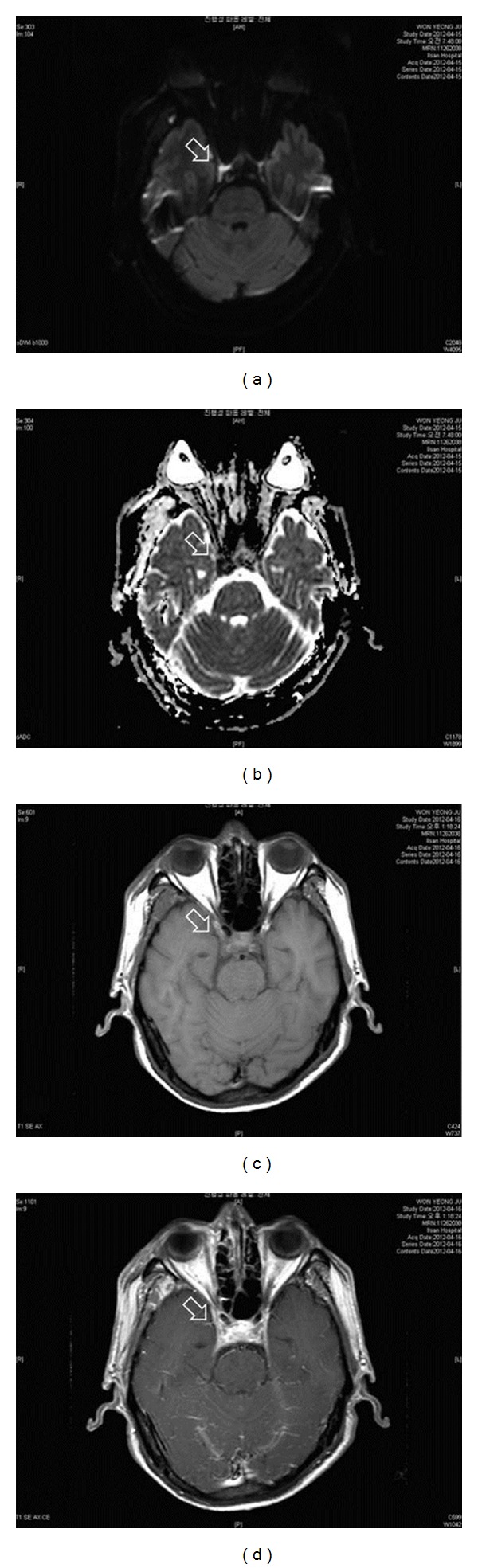
MR images reveal the presence of an abscess in the cavernous sinus area (white arrow, (a)–(d)). Diffusion restriction around the right cavernous sinus indicates an abscess in the diffusion-weighted MR image (a). There is no high signal around the right cavernous sinus in the apparent diffusion coefficient map (b). Precontrast T1-weighted MR image (c). More enhancement around the right cavernous sinus area was observed in the postcontrast T1-weighted MR image (d).

For emergency treatment of the abscess in the right pterygomandibular space, an intraoral incision was made for pus drainage. Contrast-enhanced brain magnetic resonance (MR) imaging, brain and neck MR angiography, and gadolinium imaging studies were performed the following day to evaluate the oculomotor nerve. A signal change was detected on the MR images in the right cavernous sinus area about the presence of an abscess in the cavernous sinus area (Figure 2). With the help of CT, MR images, and clinical evaluation, we were finally able to diagnose the patient with ONP caused by pterygomandibular infection originating from the lower right third molar.

**Figure 3:**
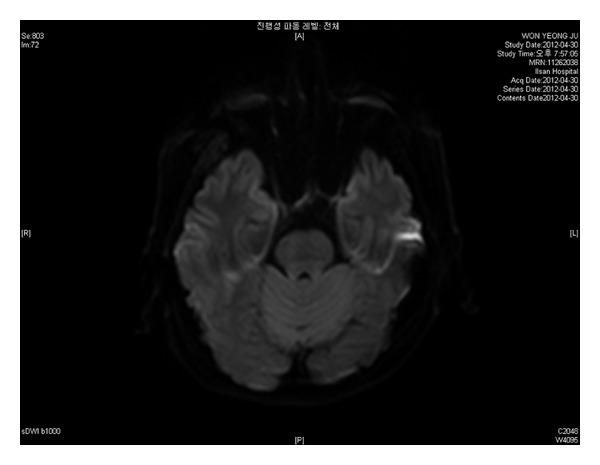
Diffusion-weighted MR image of the patient after neurological symptoms improved. Lack of diffusion restriction around the right cavernous sinus indicates that there is no residual infectious lesion.

On the second day of hospitalization, the patient's clinical symptoms of ONP, including pupil dilation and blepharoptosis, had improved. A spinal tap for cerebrospinal fluid cytology was negative. On the fourth day of hospitalization, the right lower third molar, which was thought to be the cause of the infection, was extracted and curettage was performed on the extraction site. On the ninth day in the hospital on antibiotic therapy, the patient's condition had improved and he was discharged in a state of mild right blepharoptosis after ophthalmologic and systemic evaluation, including MR images (Figure 3). No complications developed during a follow-up period of 6 months.

## 3. Discussion

ONP has various causes, including brain tumours, lesions in the orbital area, infection, and trauma [1, 2]. Microvascular risk factors for moderate or severe ONP include age, hypertension, diabetes mellitus, and a history of smoking [3].

Regarding clinical signs, ONP can cause deflection or weakness in raising the eye, introversion of an eye with accompanying blepharoptosis or mydriasis, and blurred or double vision [4]. Imaging, such as contrast-enhanced CT, angiography, or MR angiography, may be needed to differentiate ONP from an aneurysm or tumour [5]. The lesion in the cavernous sinus may affect other cranial nerves (IV, V, VI) [6].

In the present case, the patient had a systemic disease, specifically diabetes mellitus, and a smoking habit. The general condition of a patient, including systemic disease, should be taken into consideration during recovery from ONP. In the present case, the patient did not have double vision or pain in the orbital area. An enhanced lesion was found near the right sphenoid sinus area, extending from the border of the cavernous sinus in MR images. ONP resulting from odontogenic infections, especially in relation to the lower third molar, is rare.

In summary, an odontogenic abscess originating from the lower right third molar caused invasion of the right cavernous sinus area in a patient with diabetes and a smoking habit. Precise clinical examination and various imaging techniques were helpful for the proper diagnosis and interdisciplinary treatment of an infection under systemic evaluation.
